# Tonsillar colonisation of *Fusobacterium necrophorum* in patients subjected to tonsillectomy

**DOI:** 10.1186/s12879-015-0975-z

**Published:** 2015-07-10

**Authors:** Helena Björk, Lena Bieber, Katarina Hedin, Martin Sundqvist

**Affiliations:** Department of Otorhinolaryngology, Central Hospital, Växjö, SE-351 85 Sweden; Department of Clinical Microbiology, Central Hospital, Växjö, SE-351 85 Sweden; Department of Clinical Sciences, Family Medicine, Lund University, SE-205 02 Malmö, Sweden; Unit for Research and Development, Kronoberg County Council, SE-352 12 Växjö, Sweden; Department of Laboratory Medicine, Clinical Microbiology, Faculty of Medicine and Health, Örebro University Hospital, Örebro University, SE-701 82 Örebro, Sweden

**Keywords:** *Fusobacterium necrophorum*, Tonsillectomy, Recurrent tonsillitis, Chronic tonsillitis, Persistent sore throat syndrome, Aetiology, Treatment

## Abstract

**Background:**

*Fusobacterium necrophorum* is a well-known cause of Lemirre’s disease and accumulating evidence support its pathogenic role in peritonsillar abscess while its role in recurrent and chronic tonsillitis is uncertain. The objective of this study was to assess the prevalence of oropharyngeal colonisation with *F. necrophorum* and Beta-haemolytic streptococci in a cohort of patients scheduled for tonsillectomy due to recurrent or persistent throat pain, and to evaluate the dynamics of colonisation with repeated sampling during a follow-up time of 6 to 8 months.

**Methods:**

Fifty-seven (57) patients aged 15–52 years scheduled for tonsillectomy due to chronic/recurrent tonsillitis or recurrent peritonsillar abscess were included. Throat swabs for the detection of *F. necrophorum* and Beta-haemolytic streptococci and clinical data was collected at inclusion, at the time of surgery and 6 to 8 months after surgery. Statistical analysis was performed using the Chi-square, Fisher’s exact and Mc Nemar tests.

**Results:**

*Fusobacterium necrophorum* was found in 28, 30 and 16 % of the patients at inclusion, surgery and follow up respectively. The corresponding results for beta-haemolytic streptococci were 5, 9 and 5 %. Patients colonised with *F. necrophorum* at follow-up, after tonsillectomy, were equally relieved from their previous throat pain as non-colonised patients. Looking at individual patients, the culture results for *F. necrophorum* varied over time, indicating a transient colonisation.

**Conclusion:**

*Fusobacterium necrophorum* was frequently found in throat cultures in this cohort of patients with recurrent or chronic throat pain leading to tonsillectomy. Colonisation was equally frequent in the asymptomatic cohort post-tonsillectomy, indicating that *F. necrophorum* is not alone causative of the symptoms. In an individual perspective, colonisation with *F. necrophorum* was transient over time.

## Background

Recurrent or persistent sore throat is a significant health problem mainly affecting adolescents and young adults. Recurrent tonsillitis (RT), chronic tonsillitis (CT) and persistent sore throat syndrome (PSTS) are sometimes overlapping diagnoses used for this patient group. The definition of RT includes repeated bouts of acute tonsillitis (AT) within a time span of less than a few years [1–3] and the diagnoses CT and PSTS both refer to a condition with recurrent or persisting throat symptoms of lower intensity. In severe or protracted cases, patients suffering from RT, CT and PSTS are often subjected to tonsillectomy. Peritonsillar abscess is a purulent infection between the tonsillar capsule and the pharyngeal constrictor muscle regarded as a complication of acute tonsillitis [4]. Relapse of this condition is another indication for tonsillectomy according to national Swedish guidelines [5].

Despite being one of the most common causes of surgical intervention in young adults, the aetiology of RT and CT/PSTS is insufficiently researched. Beta-haemolytic *Streptococcus* group A (GAS) is considered the main bacterial pathogen in AT [6], and recurrent or relapsing infection with GAS is regarded an important cause of RT [7]. However, the aetiology of RT is more complex as other bacteria have been proposed to play a pathogenic role [8, 9], and formation of biofilms [10] as well as alterations of the normal flora [11] have been suggested to be important for the relapsing nature of the condition.

During the last decade, the anaerobic, pleomorphic Gram-negative rod *Fusobacterium necrophorum* has been suggested to play an important pathogenic role in acute and recurrent tonsillitis as well as in persistent sore throat syndrome [12–15]. It has likewise been put forward as a major pathogen in peritonsillar abscesses [4, 16]. *Fusobacterium necrophorum* has since the 1930’s been known to cause the life-threatening septic disease Lemierre’s syndrome, but it has also been considered a part of the normal human oropharyngeal flora [17]. Some recent studies [12, 18, 19] have however not been able to detect the bacterium in throat swabs and tonsil core biopsies of asymptomatic subjects, while others [14, 20, 21] have reported a carriage rate of 3.5–21 % in healthy controls.

In order to investigate the dynamics of oropharyngeal colonisation with *F. necrophorum* in patients with severe and protracted throat symptoms, we performed a longitudinal cohort study with a focus on this bacterium in patients subjected to tonsillectomy.

The aim of the study was to assess the prevalence of *F. necrophorum* and beta-haemolytic streptococci in throat swabs of patients scheduled for tonsillectomy because of recurrent tonsillitis, peritonsillar abscess or chronic tonsillitis/recurrent sore throat syndrome, and to compare this with the prevalence in the same patients at the time of surgery and 6 to 8 months postoperatively.

## Methods

A prospective cohort study was performed in the setting of Växjö Central Hospital’s ENT-department, a secondary care facility in southern Sweden serving a population of approx. 126 000. 61 patients, aged >15 years, scheduled to undergo tonsillectomy due to infectious tonsillar disease, were included between January 2011 and March 2012 (14 months). Written informed consent was obtained from all patients or, if patients were under 18 years of age, from a parent. Indications for tonsillectomy were: more than one peritonsillar abscess, recurrent tonsillitis with symptoms interfering with normal daily activities or chronic tonsillitis/persistent sore throat syndrome with symptoms interfering with normal daily activities. The latter entity was for study purposes defined as recurrent or persistent sore throat without well-defined bouts of acute tonsillitis and is hereafter called chronic tonsillitis (CT). All included patients filled in a questionnaire regarding their history of throat pain and antibiotic treatment during the preceding year. The attending otorhinolaryngologist filled in a questionnaire including previous medical history, indication for tonsillectomy and, if applicable, current infection and antibiotic treatment. Throat swabs (Amies transport medium, Copan, Italy) were obtained from all patients by the including physician following a standardized routine where the swab was rubbed on the surfaces of both tonsils. At the day of tonsillectomy, a new throat swab was obtained by the operating otorhinolaryngologist after the induction of anaesthesia. Finally, all included patients were offered a control visit 6–8 months postoperatively. All control visits were conducted by the primary investigator (HB). At this visit the patients filled in a new questionnaire regarding throat symptoms and a third throat swab was obtained, now from the tonsillar fossae. Samples were intended for study purposes only and the physician was not informed of the results. If needed, the treating physician had the possibility to take another swab for clinical decision-making.

All samples were stored in fridge until transport to the Department of Clinical Microbiology, Central Hospital, Växjö. Throat swabs were cultured for the recovery of beta-haemolytic streptococci (Lancefield group A, C and G), using a double layered agar (Columbia agar [Oxoid, Basingstokes, UK] covered with sheep Blood Agar, Blood agar base [Merck, Darmstadt, Germany] with 5 % sheep blood [SVA, Uppsala, Sweden] and 5 mg/ml methyl violet [Merck, Darmstadt, Germany] with a bacitracin disc (0.2 IU)). For *F. necrophorum* a selective anaerobic agar (Fastidious anaerobe agar [Lab M] with 5 % horse blood [Håtuna Lab AB, Bro, Sweden] supplemented with vancomycin 2.5 mg/L [ICN Biomedicals] and nalidixic acid 5.0 mg/L [MP biomedicals]) and with a kanamycin tablet 500 μg (Rosco, Taastrup, Denmark) was used. The agar plates were incubated under anaerobic conditions in 35–37 °C for 1 and 4 days respectively. For species identification, Streptex (Remel Europe Ltd. Dartford, England) and MALDI-TOF (Microflex™ mass-spectrometer and Biotyper 3.1 software, Bruker Daltonics, Bremen, Germany) was used. Only large colony variants of Beta-hemolytic streptococci were further analysed to allow a relevant group-classification.

SPSS 20.0 software for Windows was used for all statistical analyses. For descriptive statistics, median values and proportions were used. Comparisons between proportions of categorical variables in two independent groups were performed using the Chi-square test or Fisher’s exact test (two-sided), when expected frequencies were small. When the variables were considered dependent, the Mc Nemar test was used. P-values ≤ 0.05 were considered statistically significant.

The study was approved by the Regional Ethics Board in Linköping, Dnr 2010/267-31.

## Results

### Inclusion

Sixty-one patients were included in the study. Of these, four were excluded because of insufficient inclusion data. Resulting 57 patients were aged 15–52 years, median age 19. Forty-two were female and 15 male. Half the group (*n* = 28) had CT as their only indication for tonsillectomy while the other half had RT, PTA or a combination of the diagnoses (Table 1). At inclusion, 8 patients were on treatment with antibiotics due to acute infections. (PTA *n* = 5, AT *n* = 2 and infected umbilical piercing *n* = 1). Three patients were diagnosed with tonsillar infection at inclusion (PTA *n* = 1, AT *n* = 2) and received antibiotic treatment after sampling. An additional 2 patients received antibiotics (clindamycin) as part of the treatment for RT, despite paucity of current infectious signs.

**Table 1:** Culture results at inclusion in relation to baseline data

	Total *n* = 57	*F. necrophorum n* = 16	Beta-haemolytic streptococci *n* = 3	Culture-negative *n* = 38
Age 15–20	32	12	3	17
21–25	13	3	0	10
26–30	7	0	0	7
>30	5	1	0	4
Sex Male	15	4	0	11
Female	42	12	3	27
Indication for TE				
Recurrent Tonsillitis (RT)	14	4	0	10
Peritonsillar Abscess (PTA)	4	2	0	2
Chronic Tonsillitis (CT)	28	8	2	18
RT + PTA	3	0	0	3
PTA + CT	1	1	0	0
RT + CT	6	1	1	4
Other^a^	1	0	0	1
Current PTA at inclusion	6	3	0	3
Current AT at inclusion	4	2	1	1
On-going antibiotic treatment at inclusion	8	4	0	4
History of mononucleosis	13	1	2	10
Tonsillar hypertrophy	25	9	2	14

^a^One patient with a previous episode of severe acute tonsillitis

*Fusobacterium necrophorum* was detected in 16 of 57 throat swabs (28 %) and beta-haemolytic streptococci in three (5 %), one of which was group A streptococci (GAS) and two group G streptococci (GGS) (Table 2). No throat swab showed growth of more than one of the mentioned bacteria. Of the ten patients presenting with acute tonsillar infection at inclusion, six had a positive culture for *F. necrophorum* and one had growth of GAS. No significant correlations were shown between a positive culture for *F. necrophorum* and tonsillar hypertrophy (9/16 vs 16/41 *p* = 0.24 Chi square test) or self-reported history of mononucleosis (1/16 vs 12/41 *p* = 0.08 Fishers exact test). Baseline characteristics did not differ between culture positive and culture negative patients (Data not shown).

**Table 2:** Results of throat cultures per main diagnosis at different sampling times

	Recurrent tonsillitis (RT) (*n* = 20)	Peritonsillar abscess (PTA) (*n* = 8)	Chronic tonsillitis (CT) (*n* = 28)	Total (*n* = 56)^a^
Inclusion (*n* = 56^a^)	5/1/14	3/0/5	8/2/18	16/3/38
Surgery (*n* = 47)	6/0/10	2/1/4	6/3/17^b^	14/4/31^b^
Follow-up (*n* = 43)	2/1/10	1/1/5	4/0/19	7/2/34

(*F.necrophorum* /beta-haemolytic streptococci/negative)

^a^One additional patient included because of a history of severe tonsillitis had a negative culture at inclusion and denied surgery, lost to follow-up

^b^2 samples were positive for both *F. necrophorum* and beta-haemolytic streptococci

Further, the frequency and severity of throat pain, associated febrile disease and number of prescribed antibiotic treatments the last year was similarly not associated with the presence of *F. necrophorum* or beta-haemolytic streptococci at baseline (Data not shown).

### Sampling at the time of surgery

Tonsillectomy was performed on 55 of the scheduled 57 patients, while 2 patients ultimately declined surgery. The operation was performed 1–132 days after inclusion (median 44 days). Perioperative throat swabs were obtained from 47 patients. On the day of tonsillectomy, 7 out of the 16 previously culture positive patients were positive for *F. necrophorum.* Another 7 patients had growth of *F. necrophorum* despite being culture negative at inclusion (Table 3). Two patients had concomitant growth of *F. necrophorum* and beta-haemolytic streptococci (one group C streptococci, GCS, and one GGS) and 2 patients were positive for beta-haemolytic streptococci only (one GCS and one GGS). To summarize, *F. necrophorum* was found in 14/47 (30 %) and Beta-haemolytic streptococci in 4/47 (9 %) of patients (Table 2).

**Table 3:** Patients positive for *Fusobacterium necrophorum.* Culture results from three different samplings, visualizing the dynamics of colonisation in individuals. Patients were scheduled for tonsillectomy due to peritonsillar abscess, recurrent tonsillitis or chronic tonsillitis

Inclusion	Surgery after 1–132 days	Follow-up after 6 months
		Positive *n* = 3
	Positive *n* = 7	Negative *n* = 1
		Lost to follow up *n* = 3
		Positive *n* = 0
Positive *n* = 16	Negative *n* = 6	Negative *n* = 4
		Lost to follow up *n* = 2
		Positive *n* = 0
	Culture not obtained *n* = 3	Negative *n* = 3
		Lost to follow up *n* = 0
		Positive *n* = 1
	Positive *n* = 7	Negative *n* = 3
		Lost to follow up *n* = 3
		Positive *n* = 3
Negative *n* = 41	Negative *n* = 27	Negative *n* = 20
		Lost to follow up *n* = 4
		Positive *n* = 0
	Culture not obtained *n* = 7	Negative *n* = 5
		Lost to follow up *n* = 2

### Sampling at the time of follow-up

Forty-three of the 57 included patients (75 %) showed up for a follow-up visit 6 to 8 months postoperatively. All but one considered themselves cured. Seven out of these 43 patients (16 %) were positive for *F. necrophorum* (Table 2). Out of those seven, three had not had a positive culture for *F. necrophorum* before, while three had had positive cultures both at inclusion and surgery and one only at surgery (Table 3). Two patients (5 %) had positive cultures for beta-haemolytic streptococci, one of which was GCS and one GGS.

In order to compare the prevalence of *F. necrophorum* at inclusion with the prevalence at follow up, a Mc Nemar analysis was performed, showing no statistically significant difference. Only patients sampled at both occasions (*n* = 43) were included in this analysis. (11/43 vs 7/43 *p* = 0.39, Mc Nemar test).

## Discussion

In this study, throat cultures for *F. necrophorum* and beta-haemolytic streptococci were obtained pre-, per- and postoperatively from a cohort of patients scheduled for tonsillectomy due to infectious tonsillar disease. To our knowledge, this is the first study with a longitudinal approach to assess the prevalence of *F. necrophorum* in the same subjects over time. It is also the first study aimed to compare the prevalence of *F. necrophorum* before and after tonsillectomy in a cohort of patients. We found a high prevalence of *Fusobacterium necrophorum* both pre- and post-tonsillectomy in our selected patient cohort.

The studied cohort had a female preponderance and a median age of 19 years, which is representative for this group of patients according to the National Tonsil Surgery Register in Sweden (Stahlfors, pers comm). The number of included patients was lower than estimated, rising doubts about the representativity of the cohort. To address this question, a review of all tonsillectomies performed at Växjö Central Hospital during the time of the study showed that 80 % of patients meeting inclusion criteria had been included. This was considered an acceptable inclusion rate, even though a larger number of included patients would have been preferable. Seventy-five per cent of patients completed all three visits despite the high geographical mobility of the affected age group and the lack of clinical advantages associated with the follow-up visit. The drop-out rate was thus likewise considered acceptable.

Tonsil core biopsies and PCR-based techniques have been advanced as more sensitive methods for the detection of *F. necrophorum* than the surface swabs and selective culture used in this study [14, 22]. Considering that core biopsies are not an option in the clinical reality, and because they would not have been possible to compare with pre- or postoperative results, we used surface swabs for sampling. Regarding detection by PCR, it is not clear whether the increase in sensitivity increases the predictive value of a finding of *F. necrophorum* in the clinical setting. We chose selective culture as this is the method used in our clinical everyday practice.

We found a high prevalence of *F. necrophorum* in this cohort, like in previous studies of similar patient groups [4, 13, 19]. Among patients with acute infection at the time of sampling, *F. necrophorum* was found in an even higher proportion, consistent with earlier studies showing that *F. necrophorum* is frequently found in peritonsillar abscess and acute tonsillitis in adolescents and young adults [4, 13, 16, 21]. Notably, the finding of *F. necrophorum* varied at different times in the same individual, suggesting that oropharyngeal carriage of this bacterium is transient. Some patients acquired the bacterium during the follow-up time while others, initially colonised, lost it. This finding should be interpreted with some caution, as PCR-based detection and or a more standardised sampling method might have shown residual colonisation with *F. necrophorum* in lower numbers. Kissing contacts have previously been suggested as a risk factor for carriage of *F. necrophorum* [20] and a partner could thus be an important source of re-colonisation.

Interestingly, the prevalence of *F. necrophorum* in the cohort was equal at all three sampling times. Even at follow-up, when all patients but one were cured from their throat symptoms, *F. necrophorum* was found in 16 % of patients. The lack of correlation between symptomatology and presence of *F. necrophorum* in postoperative swabs suggests that the bacterium loses its pathogenic potential in the absence of tonsillar tissue. Another possible interpretation is that *F. necrophorum* does not play a pathogenic role in this group of patients, but is found merely as an asymptomatic colonisation. The causality may also differ between patients in the cohort depending on their indication for tonsillectomy. Indeed, previous studies have shown *F. necrophorum* to be an important pathogen in peritonsillar abscesses [4, 16]. This has been further supported by a recent Danish study showing that patients with growth of *F. necrophorum* in aspirates from peritonsillar abscesses also develop specific serum antibodies against the bacterium [23]. However, while growth of *F. necrophorum* in throat swabs is a common finding in recurrent and chronic tonsillitis, serological evidence is lacking for a causal role in these conditions [23].

Similarly to the epidemiology of GAS in children, we hypothesize a transient oropharyngeal colonisation of *F. necrophorum* in adolescents and young adults with the potential to cause infection under certain circumstances. Concurrent infection with Epstein-Barr virus (EBV) facilitating tissue penetration of the tonsillar epithelium and inducing a transient decrease in T-cell mediated immunity has been suggested such a circumstance [24–26]. In our study, no patient showed signs of mononucleosis at any time during the study and the aspect of co-infection is therefore not possible to evaluate. Self-reported history of mononucleosis was in our study not significantly associated with presence of *F. necrophorum* in throat swabs.

We suggest that presence of tonsillar tissue is a prerequisite for infectivity by *F. necrophorum.* The bacterium is evidently able to colonise oropharyngeal membranes of tonsillectomized individuals, but it might lack ability to cause clinical symptoms in the absence of tonsillar tissue. Notably, very few patients were colonised with beta-haemolytic streptococci at all sampling times.

## Conclusions

In conclusion, we found a high prevalence of *Fusobacterium necrophorum* both pre- and post-tonsillectomy in our selected patient cohort. Importantly though, *F. necrophorum* was not found in all patients and not always associated with symptoms. Looking at individual patients, the presence of *F. necrophorum* in the oropharyngeal flora was transient.

Since *F. necrophorum* is found in a wide clinical spectrum from asymptomatic carriage via chronic throat symptoms to acute and sometimes severe infections, a positive throat culture or molecular test for *F. necrophorum* is a true challenge for the clinician. The lack of evidence-based guidelines on the management of *F. necrophorum* in tonsillar disease warrants further research, both on its pathogenic importance and on the benefits of treatment in other clinical situations than Lemierre’s syndrome.

## Abbreviations

RT: Recurrent tonsillitis
CT: Chronic tonsillitis
PSTS: Persistent sore throat syndrome
AT: Acute tonsillitis
PTA: Peritonsillar abscess
GAS: Group A streptococci
GGS: Group G streptococci
GCS: Group C streptococci
PCR: Polymerase chain reaction
EBV: Epstein-Barr virus
